# Sociodemographic Variations in the Availability of Urban Green Spaces in an Older Swedish Population

**DOI:** 10.3390/ijerph191912651

**Published:** 2022-10-03

**Authors:** Kristoffer Mattisson, Anna Axmon, Gunilla Carlsson, Agneta Malmgren Fänge, Connie Lethin, Emilie Stroh

**Affiliations:** 1 Division of Occupational & Environmental Medicine, Lund University, 223 62 Lund, Sweden; 2 EPI@LUND (Epidemiology, Population Studies, and Infrastructures at Lund University), Lund University, 223 62 Lund, Sweden; 3 Department of Health Science, Lund University, 222 40 Lund, Sweden

**Keywords:** ageing, cross-sectional, geographical information systems, neighborhood, socioeconomic, urban green space

## Abstract

Urban green spaces (UGS) can have a positive impact on health and thereby potentially ease the strain on the health care system. However, the availability and benefits seem to vary between different sociodemographic groups. The aim of this study was to investigate associations between sociodemographic factors and availability to UGS among people aged 65 years or older. Data on sociodemographic variables and residential coordinates were obtained for three cross-sectional cohorts in two cities (Malmö and Kristianstad) and three years (2010, 2015, and 2019). Three measures of urban green spaces; total (UGS), public (PGS) and quiet (QGS), within 300 m were used to determine availability. The results indicated higher availability to both total and publicly available urban green spaces for groups with lower socioeconomic status (SES) is positive from a health perspective. However, availability to high qualitative publicly available urban green spaces, from a noise perspective, was lower, indicating the opposite.

## 1. Introduction

Worldwide, an increasing proportion of the population is living in urban areas, resulting in both expansion in size of urban areas and densification [1]. As cities are growing and densifying, the competition for space increases. This has led to a decrease of urban green spaces (UGS) in Europe, and even more so in Asia and Australia [1]. In Sweden, the trend of urbanization has been clear. Today, almost nine out of ten Swedes live in urban areas, whereas the opposite was true 200 years ago [2].

There are only a few studies measuring changes in UGS, in this study defined as any vegetation found in the urban environment [1], using methodology that enables comparisons over time have been made for Swedish cities. Statistics Sweden found a reduction in UGS by 0.6–1.5% in the ten largest cities (including Malmö) in Sweden from 2000 to 2005 [3]. However, a later study including only Stockholm found an increase in Normalized Differentiated Vegetation Index (NDVI), i.e., vegetation cover, between 1990 to 2015 [4].

It is well known that UGS can provide so-called ecosystem services (for example, regulate temperature, produce biomass, and reduce risk of flooding) and improve biodiversity inside cities, as well as have a positive impact on health and wellbeing on residents [5]. A growing literature on positive effects of UGS on health includes reduced risk of cardiovascular disease [6], lower mortality [7,8] as well as mental health and wellbeing [9,10]. The promotion of physical activity from availability to UGS is of importance, but also merely viewing it from a window can affect physiological health [11]. Thus, the benefit of a green environment can be due to experiencing the nature as well as using it for, e.g., physical activity. A range of factors impact the use of UGS, such as experience of safety [12] and walkability [13]. However, a basic condition for partaking is access. Given that a majority of people live in urban areas, an uneven distribution of UGS could contribute to environmental injustice and consequently increase already existing health gaps between different sociodemographic groups. However, UGS could also potentially help closing these gaps if otherwise disadvantaged groups have greater availability.

Several studies report disparities in the associations between UGS and health for different sociodemographic groups, i.e., UGS may be more beneficial to some groups than others [14,15,16,17]. A systematic review on sex and gender differences in associations between UGS and physical health (including cardiovascular disease, diabetes, and mortality) found a stronger protective effect for these outcomes in general for women than for men [17]. This indicates that women have more to gain from having available UGS than men. The effect was particularly strong when considering UGS within a maximum of 500 m from the residency. Other disadvantaged groups, such as those with low-income or belonging to ethnic minorities, have also been found to have more beneficial health effects from UGS, especially those publicly available such as parks [15]. Moreover, housing type have been found to modify the association between UGS and health [14]. In an Australian study more UGS (including trees, scrubs, and open grass) within a 1.6 km road network analysis was associated with less physiological distress among single-family house-dwellers, while no such association were found for those living in apartments. When considering only tree canopies, less physical distress was found also for those living in apartments. Only when considering grassland, more physiological distress was found for both groups.

Sociodemographic inequalities in the association between UGS and health can also interact with other sociodemographic factors. In a study from Brussels investigating self-perceived health and mortality, low educated women had the strongest beneficial effect on health from UGS, while among men those with higher education had the strongest positive effect [16].

Availability to UGS has been found to be unevenly distributed between different sociodemographic groups based on, e.g., ethnicity and income [18,19,20], which be referred to as environmental injustice [21]. A review paper from the World Health Organization (WHO) reported that in ecological studies, deprived areas had lower availability of UGS, while the associations were mixed for studies on individual level [22].

To maintain good health over the life course, it is important to continue to exercise physical activity [23]. Prior studies have found positive associations between availability to UGS and physical activity among older people [24,25]. They are also important for good health is social interactions, and older adults’ availability to UGS has been found to promote social ties [26]. In addition, after retirement, the amount of time that we spend at home or in close vicinity to our residence could be expected to increase, but also the time spent in close vicinity of our residence. It could therefore be argued that availability to UGS, especially close to the residence, becomes even more important with increasing age. The aim of this study was to investigate variations in availability to UGS based on sociodemographic groups among people aged 65 years or older in Sweden. In the present study, we define UGS as any land covered by vegetation found in the urban environment [1].

## 2. Materials and Methods

### 2.1. Study Area

**Figure 1:**
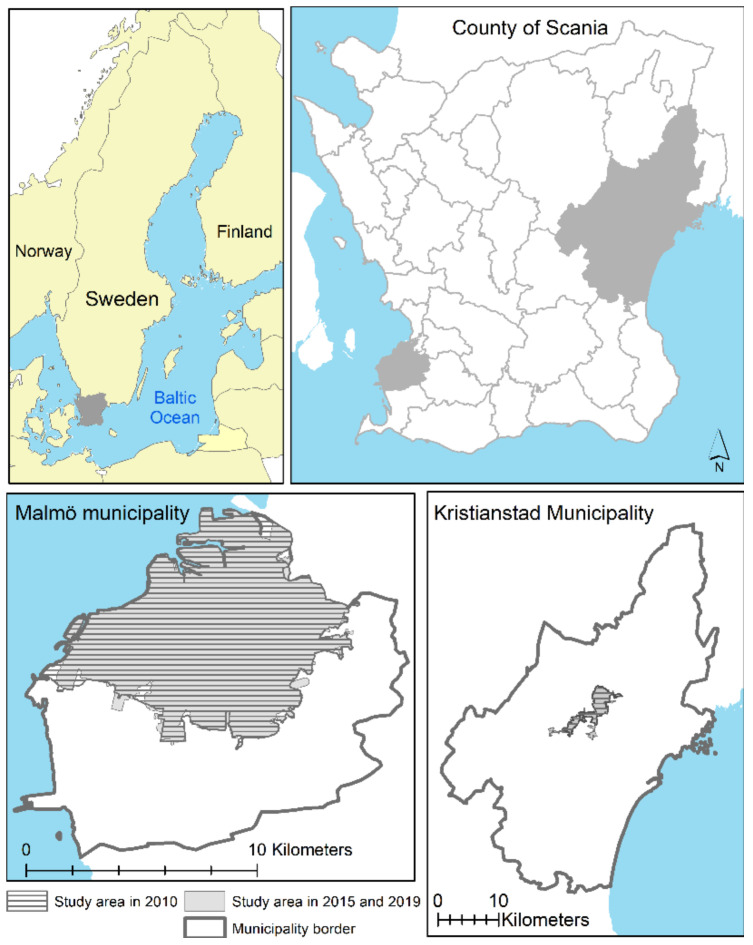
Overview map of location of the county of Scania in Sweden (upper left), the municipalities Malmö and Kristianstad location in Scania (upper right) and study areas in Malmö (lower left) and Kristianstad (lower right) for the three cohorts.

The study area was the urban areas of the two cities Malmö and Kristianstad, located in Scania, the southernmost county of Sweden (Figure 1).

Based on population size, Malmö is the largest municipality in Scania with about 299,000 inhabitants in 2010 and 344,000 in 2019 [27]. It is located at the coast of Öresund and in the middle of an area with some of the most valuable agricultural land in Sweden. It is directly connected to Copenhagen (the Danish capital) via the Öresund bridge. The city of Malmö is rapidly growing and has a higher population density (2193 inhabitants/km^2^) [27] than both Gothenburg (the second-largest city in Sweden) and Stockholm (the Swedish capital and largest city). There is a political ambition to continue to densify the city and the municipality [28].

Kristianstad is located in the northeast corner of Scania. It was selected as a study area to represent a smaller and less densely populated municipality (69 inhabitants/km^2^), and thereby a different geographical context. Kristianstad had about 79,500 inhabitants in 2010 and 85,700 in 2019 [27].

The city of Malmö and Kristianstad, as identified by Statistics Sweden, within respectively municipality were included in this study. However, as Statistics Sweden’s definition of urban areas can vary over time and, the extent of the study area was slightly different in 2010 compared to 2015 and 2019 (Figure 1).

### 2.2. Study Population

This was a repeated cross-sectional study in which six cohorts were defined. These cohorts included all persons aged 65 years or older living in the city of Malmö and Kristianstad, within respectively municipality, in 2010, 2015, and 2019. After excluding people with missing data on UGS or any of the sociodemographic variables the final study population was *N* = 48,224 in 2010, *N* = 50,367 in 2015 and *N* = 53,280 in 2019 (Table 1).

**Figure 2:**
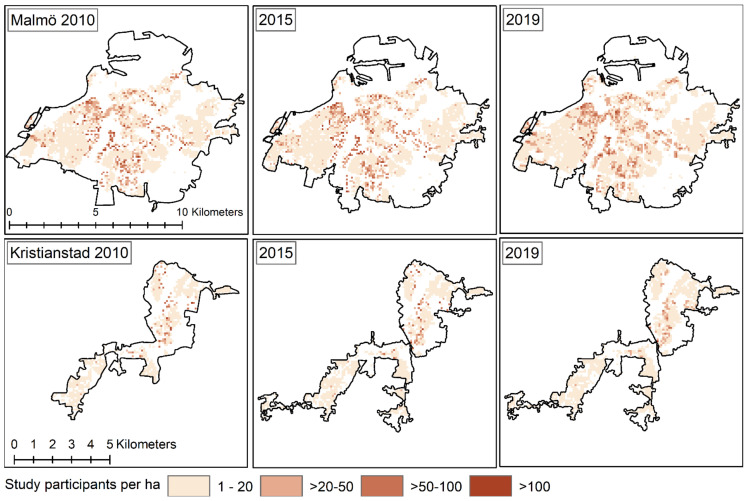
Residential density of persons 65 years or older in Malmö and Kristianstad city, within respectively municipality in 2010, 2015 and 2019.

The residential density in the cities are unevenly distributed spatially and presented in Figure 2.

### 2.3. Sociodemographic Variables

Five individual level and one area level sociodemographic variables were used. Information on individual characteristics was collected from the register of the total population at Statistics Sweden and information on residential area socioeconomic status (SES) from Statistics Sweden’s homepage (www.scb.se; accessed on 2 May 2022). Residential area was represented by Demographical statistical area (DeSO), which is administrative units with 700 to 2700 inhabitants, with varying geographical coverage. Proportion of people with low economic standard (less than 60% of the median of the total disposable income of the household) was used as a measure of SES for the residential area, for individual study participants.

Characteristics that were not inherently dichotomous were dichotomized at the overall median (i.e., the median across both municipalities and all three years). Moreover, characteristics that may change over time were determined for the start of the years investigated (31 December 2009, 2014, and 2018, respectively).

1. Age—Estimated as the difference between year of birth and study year (i.e., 2010, 2015, and 2019, respectively). Those above the median were used as reference category, i.e., older people (aged 74+ years) were compared to younger study participants (65–74 years).
2. Sex—Woman (reference category) or man
3. Housing—Living in apartment (reference category) or single-family house [house].
4. Cohabiting—Living alone (reference category) or cohabiting. This category concerns living condition rather than civil status. That is, an individual could be registered as married but live alone.
5. Place of birth—Born abroad (reference category) or born in Sweden
6. Socioeconomic status (SES) in area of residence—Those above the median (19%) were used as reference category, i.e., those living in high SES areas (areas with a low percentage of people with low SES) were compared to those living in low SES areas (i.e., areas with a high percentage of people with low SES).

### 2.4. Urban Green Spaces

**Figure 3:**
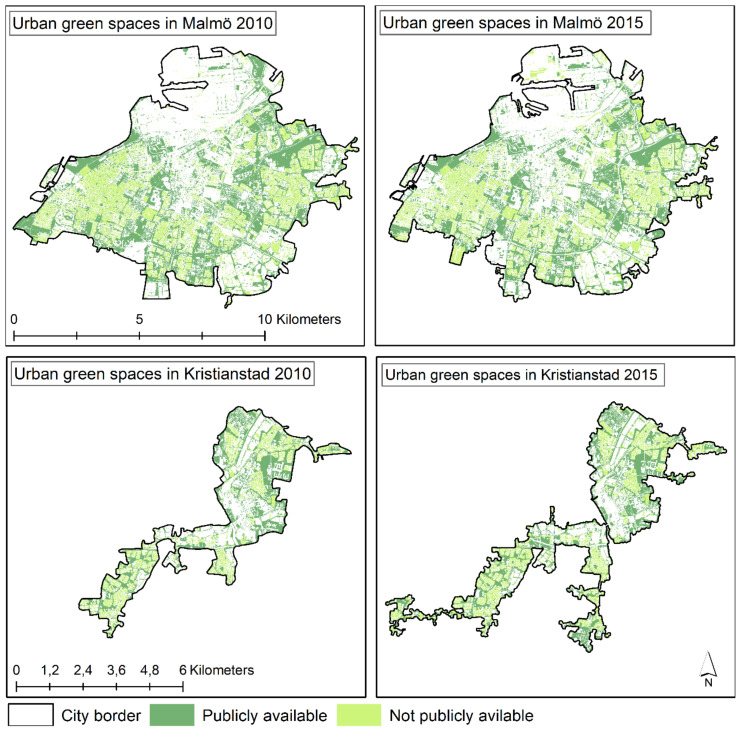
Map showing distribution of total and publicly available urban green spaces Malmö and Kristianstad city, within respectively municipality in 2010 and 2015.

To quantify the physical amount of UGS in the vicinity of the residence (i.e., the availability of UGS), Geographical Information System (GIS) data layers with land cover classifications of vegetation, compiled by Statistics Sweden, were used (see Figure 3) [29,30]. The surveys were carried out in 2010 and 2015, mapping UGS in Sweden including urban areas in Malmö and Kristianstad municipality. Similar methods were used for the classifications both years, allowing comparison between years to be made [29]. Generally, satellite images with high spatial resolution (10 × 10 m) were classified into different types of land cover. This analysis was complemented with input from a number of other GIS layers, for instance information about buildings and road network, used to make a more detailed classification of total UGS as well as publicly available UGS. The following categories of UGS were used to assess availability of UGS in this study:

Urban green spaces (UGS)—The total area within the urban area covered by green elements for instance gardens (including private gardens), parks, trees, and other grass surfaces, independent of how they are used. The exception is green roofs, which are not considered part of the ground cover.

Publicly available urban green spaces (PGS)—The part of total UGS that is publicly available, with consideration to ownership of the land. To be considered as publicly available, areas should be free to access throughout the year. Areas privately own, but covered by the right of public access, for instance pasture or meadows were considered as publicly available.

Quiet publicly available urban green spaces (QGS)—In addition to the classification from Statistics Sweden, a detailed, modelled noise levels from road traffic respectively railway noise were available for the municipality of Malmö, based on the traffic flows in 2016 [31]. Areas with noise levels below 45 dB(A) L_Aeq24_ were considered as quiet and analyzed in correspondence with publicly available UGS. This level was selected based on levels where roughly all visitors would perceive the acoustic quality as good [32].

#### Measures of Availability of Urban Green Spaces

Based on the classification of UGS from Statistics Sweden and available data on noise three measures were selected and assessed for the study population (see Table 2). A classification of UGS was not available specifically for 2019, why the classification of UGS for 2015 were used to address availability to UGS for the study population in both 2015 and 2019.

Residential addresses were known as coordinates for the centroid of the property for each study participant. As measures of availability of UGS based on these coordinates, we used percentage of UGS, PGS, and QGS within 300 m (main analyses) of the residence, respectively. The distance of 300 m has commonly been used in epidemiological studies to study both physical and mental health outcomes in relation to UGS [33]. In addition, sensitivity analyses for corresponding measured within 100 m were conducted. A shorter distance was considered as it may be more relevant for an older study population and therefore selected for the sensitivity analysis.

The different measures of urban greenness were dichotomized at the median for all three years and both cities (UGS in 300 m 51.4%, PGS in 300 m 26.6%, and QGS in 300 m 3.3%). Based on this, the outcome was having more than the median, i.e., high availability to UGS, PGS, and QGS, respectively.

### 2.5. Statistical Analysis

Relative risks (RRs) with 95% confidence intervals (CIs) for having more than the median green space (i.e., high availability) within 300 m (primary analysis) and 100 m (sensitivity analysis) were estimated for UGS, PGS, and QGS using generalized linear models with Poisson distribution and log link. Each sociodemographic variable was analyzed separately (crude estimates) as well as in a model with all sociodemographic variables included (adjusted estimates). As the different outcome measures were not normally distributed, pairwise correlations were determined using Spearman’s correlation coefficient.

All analyses were done using IBM SPSS Statistics 27.0. *p*-values below 0.05 were considered statistically significant.

## 3. Results

### 3.1. Distribution of Sociodemographic Variables

The distribution of sociodemographic variables generally differed between Malmö and Kristianstad city, in that people in Malmö city were more likely to live in an apartment, live alone, be born abroad and live in an area with low SES (except for 2010; see Table 3). However, within each city, the patterns were similar for all three years.

### 3.2. Overall Availability of UGS, PGS and QGS

Overall, the study population in Kristianstad city had high UGS availability compared to those in Malmö city, except in 2010 where the opposite was true (see Table 4). The same pattern was found for PGS comparing the two cities. Only a small proportion of the PGS was quiet.

Using a 300 m buffer, positive correlations were found between UGS and PGS (r_S_ = 0.552), where roughly half of the variance could be explained, and between PGS and QGS (r_S_ = 0.149). In contrast, a negative correlation was found between UGS and QGS (r_S_ = −0.045). High correlations were found between 100 m and 300 m buffer for UGS (r_S_ = 0.787), PGS (r_S_ = 0.655), and QGS (r_S_ = 0.695).

### 3.3. Availability of UGS, PGS and QGS in Different Sociodemographic Groups

**Figure 4:**
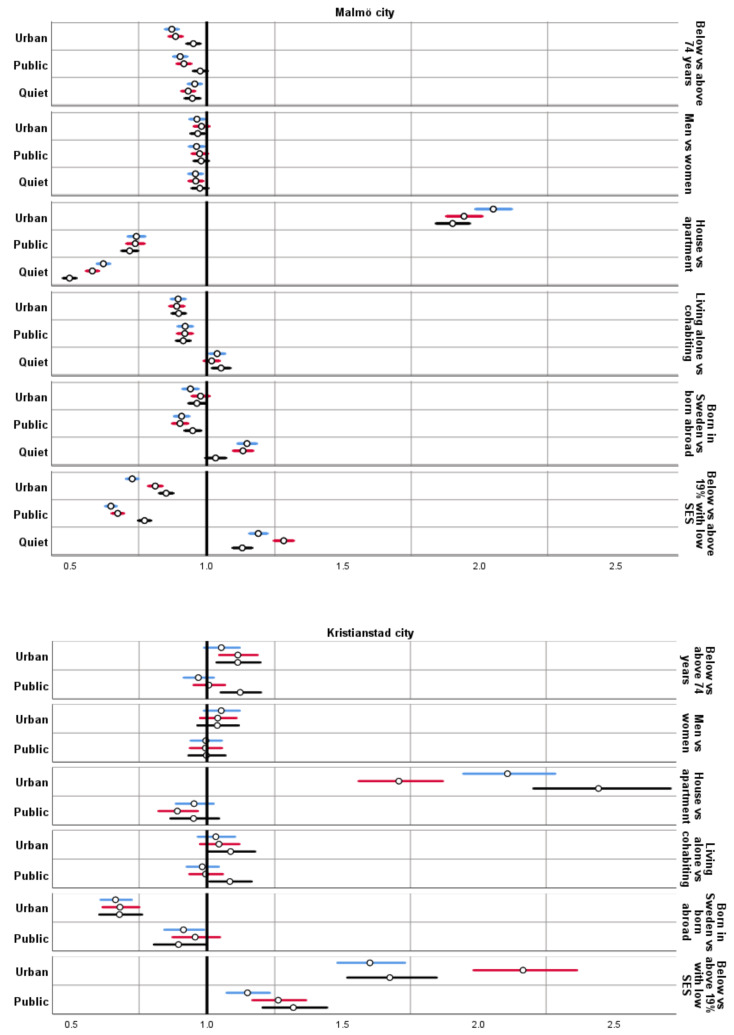
Adjusted risk ratios (dots) with 95% confidence intervals (lines; blue = 2019, red = 2015, black = 2010) for associations between sociodemographic variables and different measures of urban green spaces within 300 m of residency in Malmö city (top) and Kristianstad city (bottom).

The results for adjusted RRs comparing availability (defined as having more than the median within the buffer) of UGS, PGS and QGS within 300 m for different sociodemographic groups are presented below (Figure 4). For the estimates from the crude models and sensitivity analysis for 100 m, see Appendix A (Table A1, Table A2, Table A3, Table A4, Table A5 and Table A6).

#### 3.3.1. Age

Older age (74+ years) was associated with higher availability of all three measures of urban greenspaces (UGS, PGS and QGS within 300 m) in Malmö, and lower availability of UGS within 300 m in Kristianstad.

#### 3.3.2. Sex

There were no major differences between men and women in any of the measures of availability to UGS within 300 m, although men in Malmö had slightly lower availability of all three measures.

#### 3.3.3. Housing

Study participants living in single-family houses in both Malmö and Kristianstad in general had higher availability of UGS and lower of PGS within 300 m. There was also lower availability to QGS within 300 m for those living in houses in Malmö.

#### 3.3.4. Cohabiting

In Malmö, those cohabiting had higher availability of UGS and PGS within 300 m. The opposite was found for Kristianstad, although only a few of the estimates were statistically significant.

#### 3.3.5. Place of Birth

Those born abroad had higher availability to UGS and PGS within 300 m in both cities. In 2015 and 2019 those born in Malmö had lower QGS within 300 m available.

#### 3.3.6. Socioeconomic Status

In Malmö, those living in high SES areas (below 19% with low SES in the area) had less UGS and PGS within 300 m available. Even so, this group had higher availability to QGS within 300 m. For Kristianstad the opposite was true for UGS and PGS within 300 m.

## 4. Discussion

In Malmö, the younger old (aged 65–74 years), people living in apartments, and men consistently had lower availability of UGS within 300 m. People living alone, being born in Sweden, and residing in high SES areas, had lower availability to UGS and PGS within 300 m, but higher availability to QGS. In contrast, living in high SES areas in Kristianstad was associated with higher availability to UGS and PGS within 300 m.

Overall, the results found within Malmö and Kristianstad city were stable across model (crude and adjusted) and for all years for most associations between all measures of UGS within 300 m and the sociodemographic variables. The geographical context seemed to be of some importance as the associations were different for the two municipalities for some of the variables (age, cohabiting, and area level SES), but similar for others (sex, housing, and place of birth). The influence of the geographical context on these associations is complex, and most likely include differences in both the spatial distribution of UGS and sociodemographic groups.

The older part of the Malmö study population (i.e., those aged 74+, depending on year) had more of UGS, PGS, and QGS within 300 m available than those 65–74 years, respectively. As increasing age is associated with increased limitations in mobility, having more green space near your residence may be more beneficial the older a person becomes. A Chinese study on persons aged 65 years or older (with an oversampling of those aged 80 years or older) found that the quartile with the highest residential green (measured as NDVI within 500 m from the residence) had 14% lower odds of frailty then the lowest quartile [34]. This indicates a protective effect on health from availability of UGS. The high availability among 74+ in Malmö is thereby positive from a health perspective.

There was only small difference between men and women in availability of all measures of UGS within 300 m, although men in Malmö had slightly lower availability. A recent review conclude that women’s physical health is more positively affected by having availability to UGS [17]. Even so recent research from the UK has found sex difference in the protective effect from green spaces, in that men seemed to benefit more in relation to some health outcomes [35].

Those living in single-family houses had more UGS within 300 m but less PGS and QGS available. The higher availability of UGS is to be expected as private gardens are included in this measure. Moreover, it is fair to assume that city planners are more likely to place PGS and QGS in residential areas where few residents have their own garden. Even so, public parks and recreational areas may provide opportunities for physical activity, social interaction and recovery that are not possible in private gardens in the same way as the opposite can be true. Thus, high availability of UGS may not necessarily balance out low availability of PGS and QGS or vice versa.

Those living alone in Malmö had less UGS and PGS within 300 m available. This can be considered as negative from a wellbeing perspective as those living alone has been found to have a stronger protective effect against from UGS in relation to loneliness [36].

Being born abroad was associated with having more UGS and PGS within 300 m available in general. Thus, our findings did not indicate any environmental injustice in relation to country of origin, but rather the opposite. However, people born abroad were less likely to have more QGS within 300 m available. Thus, when including a qualitative measure of green space, environmental injustice was evident. A possible explanation for this is that being born abroad is a proxy of SES, and that areas with low SES in general are located closer to the ring roads in Malmö, i.e., areas where quiet space is scarce. The group of older people (65+ years) being born abroad is heterogenous and includes people from all countries other than Sweden, including, e.g., EU and other western countries. However, in Malmö in 2019, the three largest groups of non-Swedish born were from Iraq, Syria, and former Yugoslavia [27]. Thus, most of the people being born abroad in the study population can be assumed to be people from low income and/or conflict areas who have been forced to migrate and are more likely to have low SES.

In Malmö, those living in a high SES area had availability to less UGS and PGS within 300 m compared to those living in low SES areas. In contrast, in Kristianstad, with more UGS and PGS within 300 m found for those living in area with high SES. This is in line with findings from a study from Stockholm that showed a negative association with socioeconomic status and greenness (less green) for larger municipalities and the opposite for smaller ones [4]. Interestingly, in contrast to UGS and PGS within 300 m, in our study, living in low SES areas was associated with less QGS. This could potentially indicate environmental injustice when including a qualitative measure of the green space.

### 4.1. Strengths and Weaknesses

In the present study, we based all measures of greenness on amount of vegetation. We also tried to introduce quality aspects by including noise levels as well as publicly availability. Even so, these are only a few of the many aspects related to the usage and quality of UGS. Other important aspects include aesthetics and attractions, biodiversity, amenities, safety, usage, and facilities [37]. Thus, the measures used in the present study cover only a part of UGS quality. Even so, considering the complexity of actual impact on health of different qualities of UGS, including a large number of potential pathways, we believe that assessing total and publicly available UGS provides a collected measure of potential pathways, even though it does not allow us to differentiate between them.

There is currently no golden standard for measuring UGS. A recent review found that when studying associations with mental health, buffer distances of 300 m or shorter were often used, and for physical health between 300 m and 1000 m (not focusing specifically on older persons) [33]. In the present study, as the study population was 65 years or older, a buffer distance in the lower end of this range was considered as most relevant in relation to mobility (especially among the old-old). Thus, we chose to use the percentage of total, publicly available, and quiet green spaces, respectively, within a 300 m buffer from the residential address. We also included sensitivity analyses using a 100 m buffer. The correlations between 100 m and 300 m were high and the patterns of the RRs across the different measures of UGS were similar, thus strengthening the results.

Modelled noise levels were only available for 2016. Assuming that the noise levels were the same in 2010, 2015 and 2019, may cause a certain degree of misclassification. A study from Gothenburg studying historical noise levels found that noise exposure for individuals over time had increased [38], suggesting that there might be some over estimation of noise levels in 2010 and underestimation in 2019. However, the cohorts in the present study are rather close in time to modelled noise levels, so the extent of this error is most likely limited.

### 4.2. Generalizability and Future Studies

The results from this study, and similar studies, can be used as a basis for decision-making in relation to interventions for specific sociodemographic groups may be in greatest need of interventions. If doing so, it is important to consider that there is a risk of driving gentrification through increasing UGS, i.e., making neighborhoods more attractive and pushing away residents with lower SES [39].

In this study the availability of UGS, PGS and QGS within 300 m were used as measures of different types of UGS. However, further studies should include qualitative aspects to provide urban planners with better basis for decisions. Such qualities may include aspects such as serenity, wilderness, species richness, spaciousness, and cultural history [12] as well as maintenance and perceived security are also important [40]. With this said, potential pathways by which UGS can have a positive effect on health include increased physical activity, more social interaction, improved air quality and stress reduction [41]. Understanding the complexity of qualities of UGS and their importance for health requires further investigation to better be understood. Even so, we believe that the measures of different types of UGS could be used to better understand the associations between green space and health. Other important aspects could be to measure accessibility or actual usage of the UGS to provide even better basis for decision-makers.

## 5. Conclusions

Overall, among older people, availability to various type of urban green spaces differ for different sociodemographic groups in Malmö and Kristianstad. The findings of higher availability to both total and publicly available urban green spaces for groups with lower SES is positive from a health perspective. However, availability to high qualitative publicly available urban green spaces, from a noise perspective, was lower, indicating the opposite. The relatively high correlations between the buffer distances 100 m and 300 m suggest that the selection of buffer distance (at least distances of this magnitude) is of smaller importance than comparing different quantitative measures of UGS.

## Figures and Tables

**Table 1:** Selection of study population from three already existing cohorts.

	2010	2015	2019
Original cohorts	62,281	67,233	70,810
Excluded (missing info on urban green spaces)			
Missing residential coordinates	126	251	105
Living outside the city border	13,156	11,438	12,317
Excluded (missing info on sociodemographic info)			
Missing information on type of housing	129	209	195
Other type of housing than house or apartment	626	848	897
Other type of cohabitation than cohabiting or living alone	20	36	44 *
Included in analyses	48,224	50,367	53,280
Malmö city	41,702	43,161	45,752
Kristianstad city	6522	7206	7528

* One person is listed as “child” and living in house/apartment, i.e., included in two places.

**Table 2:** Measures of urban green spaces included in the study.

Measure of Availability of UGS	Description
UGS within 300 m (%)	The proportion of all urban green spaces within 300 m from the residency. ^1^
PGS within 300 m (%)	The proportion of publicly available urban green spaces within 300 m from the residency. ^1^
QGS within 300 m (%)	The proportion of publicly available urban green spaces with a noise level from traffic lower than 45 L_Aeq24_ within 300 m from the residency. ^1^

^1^ The part of the area outside the city border is excluded for those living closer than 300 m from the border.

**Table 3:** Sociodemographic characteristics of the study population in Malmö city and Kristianstad city.

		Malmö City						Kristianstad City					
		2010		2015		2019		2010		2015		2019	
		*n*	%	*n*	%	*n*	%	*n*	%	*n*	%	*n*	%
Total		41,702		43,161		45,752		6522		7206		7528	
Sex	Men	17,021	41	18,390	43	20,073	44	2642	41	3041	42	3269	43
	Women	24,681	59	24,771	57	25,679	56	3880	59	4165	58	4259	57
Housing	House	8437	20	8 948	21	9230	20	2245	34	2786	39	2863	38
	Apartment	33,265	80	34,213	79	36,522	80	4277	66	4420	61	4665	62
Cohabiting	Living alone	23,638	57	24,232	56	25,510	56	3269	50	3541	49	3796	50
	Cohabiting	18,064	43	18,929	44	20,242	44	3253	50	3665	51	3732	50
Place of birth	Abroad	9198	22	11,141	26	13,346	29	620	10	784	11	1080	14
	Sweden	32,504	78	32,020	74	32,406	71	5902	90	6422	89	6448	86
Age	<74 years	18,427	44	20,929	48	22,199	49	2853	44	3269	45	3247	43
	74+ years	23,275	56	22,232	52	23,553	51	3669	56	3937	55	4281	57
Low SES	<19%	19,720	47	21,054	49	22,328	49	2192	34	2665	37	3111	41
	19+ %	21,982	53	22,107	51	23,424	51	4330	66	4541	63	4417	59
Age	Median	75		74		74		75		75		75	
	Min/max	65/103		65/104		65/107		65/104		65/103		65/104	
	Q1/Q3	69/82		69/81		69/80		69/82		69/82		70/81	
% of people with low SES in area of residence	Median	19		19		19		22		22		21	
	Min/max	4/81		4/81		5/78		6/67		6/67		5/69	
	Q1/Q3	14/32		14/32		14/31		19/25		10/24		11/28	

**Table 4:** Availability of urban green spaces (UGS), publicly available urban green space (PGS) and quiet publicly available urban green space (QGS) within 300 m from the residency for the study population in Malmö city (*n* = 41702 in 2010, *n* = 43161 in 2015, and *n* = 45752 in 2019) and Kristianstad city (*n* = 6522 in 2010, *n* = 7206 in 2015, and *n* = 7528 in 2019).

Measure of Greenness		Malmö City			Kristianstad City		
	Year	Median	Min/Max	Q1/Q3	Median	Min/max	Q1/Q3
UGS within 300 m (%)	2010	54	2/89	42/60	50	12/75	36/60
	2015	50	1/83	36/57	56	12/89	43/64
	2019	50	1/83	33/57	55	12/88	43/65
PGS within 300 m (%)	2010	28	1/89	20/38	28	9/65	23/35
	2015	25	0/74	17/35	32	8/63	24/37
	2019	24	1/76	16/35	31	8/66	24/38
QGS within 300 m (%)	2010	3	0/45	0/6			
	2015	4	0/53	1/8			
	2019	4	0/55	1/8			

## Data Availability

Since the data comprise information about single individuals and is detailed enough to enable identification of, at least, some of the people included, they cannot be made publicly available. However, as the database was compiled by national register data, other researchers may contact the register holder (Statistics Sweden) to get access to the registries used in this study, and thereby generate an identical database.

## Appendix A

**Table A1:** Risk ratios for crude and multivariate associations between younger (65–74 years old) vs older (74+ years old) study participants in availability to total urban green space (Urban), publicly available urban green space (Public) and quiet publicly available urban green space (Quiet). RR for 100 m and 300 m buffer distances from the residence for the three different types of urban green spaces are presented.

		Malmö City						Kristianstad City					
		Crude			Adjusted			Crude			Adjusted		
		RR	Lower	Upper	RR	Lower	Upper	RR	Lower	Upper	RR	Lower	Upper
2010	Urban 100 m	0.999	0.973	1.025	**0.948**	**0.923**	**0.973**	**1.266**	**1.180**	**1.357**	**1.089**	**1.014**	**1.170**
	Urban 300 m	0.999	0.973	1.025	**0.951**	**0.926**	**0.976**	**1.286**	**1.198**	**1.380**	**1.113**	**1.036**	**1.197**
	Public 100 m	**0.959**	**0.933**	**0.985**	**0.964**	**0.938**	**0.990**	1.034	0.971	1.100	1.064	0.998	1.134
	Public 300 m	**0.974**	**0.948**	**1.000**	0.976	0.950	1.002	**1.127**	**1.057**	**1.202**	**1.122**	**1.051**	**1.199**
	Quiet 100 m	**0.960**	**0.933**	**0.987**	1.005	0.977	1.034	.	.	.	.	.	.
	Quiet 300 m	**0.911**	**0.885**	**0.939**	**0.947**	**0.919**	**0.976**	.	.	.	.	.	.
2015	Urban 100 m	**0.959**	**0.933**	**0.986**	**0.916**	**0.891**	**0.942**	**1.156**	**1.090**	**1.227**	**1.063**	**1.001**	**1.128**
	Urban 300 m	**0.923**	**0.898**	**0.949**	**0.885**	**0.861**	**0.911**	**1.228**	**1.153**	**1.307**	**1.114**	**1.046**	**1.186**
	Public 100 m	**0.961**	**0.935**	**0.989**	**0.959**	**0.932**	**0.987**	0.979	0.923	1.039	0.999	0.941	1.061
	Public 300 m	**0.920**	**0.894**	**0.946**	**0.916**	**0.890**	**0.942**	1.009	0.953	1.067	1.007	0.951	1.066
	Quiet 100 m	**0.967**	**0.942**	**0.993**	0.998	0.972	1.025	.	.	.	.	.	.
	Quiet 300 m	**0.906**	**0.882**	**0.929**	**0.932**	**0.908**	**0.956**	.	.	.	.	.	.
2019	Urban 100 m	**0.928**	**0.903**	**0.954**	**0.885**	**0.860**	**0.910**	**1.110**	**1.047**	**1.177**	1.035	0.975	1.098
	Urban300 m	**0.910**	**0.886**	**0.935**	**0.872**	**0.848**	**0.896**	**1.133**	**1.066**	**1.204**	1.053	0.990	1.120
	Public 100 m	**0.942**	**0.917**	**0.968**	**0.935**	**0.910**	**0.961**	**0.914**	**0.863**	**0.968**	**0.932**	**0.879**	**0.988**
	Public 300 m	**0.915**	**0.890**	**0.941**	**0.902**	**0.878**	**0.928**	0.974	0.921	1.030	0.968	0.915	1.025
	Quiet 100 m	0.986	0.961	1.012	1.019	0.993	1.045	.	.	.	.	.	.
	Quiet 300 m	**0.925**	**0.902**	**0.949**	**0.956**	**0.932**	**0.981**	.	.	.	.	.	.

* Statistically significant results (*p* < 0.005) are marked with bold text.

**Table A2:** Risk ratios for crude and multivariate associations between men vs women study participants in availability to total urban green space (Urban), publicly available urban green space (Public) and quiet publicly available urban green space (Quiet). RR for 100 m and 300 m buffer distances from the residence for the three different types of urban green spaces are presented.

		Malmö City						Kristianstad City					
		Crude			Adjusted			Crude			Adjusted		
		RR	Lower	Upper	RR	Lower	Upper	RR	Lower	Upper	RR	Lower	Upper
2010	Urban 100 m	**1.035**	**1.008**	**1.062**	**0.965**	**0.940**	**0.992**	**1.208**	**1.126**	**1.296**	1.033	0.961	1.110
	Urban 300 m	**1.035**	**1.009**	**1.062**	**0.967**	**0.941**	**0.993**	**1.197**	**1.115**	**1.285**	1.038	0.965	1.117
	Public 100 m	**0.971**	**0.945**	**0.997**	0.989	0.962	1.017	0.981	0.921	1.045	1.010	0.946	1.079
	Public 300 m	0.978	0.952	1.004	0.979	0.953	1.007	1.003	0.940	1.071	0.998	0.932	1.068
	Quiet 100 m	**0.919**	**0.893**	**0.945**	0.991	0.962	1.020	**.**	**.**	**.**	.	.	.
	Quiet 300 m	**0.922**	**0.895**	**0.949**	0.975	0.945	1.005	**.**	**.**	**.**	.	.	.
2015	Urban 100 m	**1.034**	**1.006**	**1.063**	**0.968**	**0.941**	**0.996**	**1.106**	**1.042**	**1.174**	1.020	0.959	1.084
	Urban 300 m	**1.040**	**1.012**	**1.069**	0.981	0.953	1.009	**1.153**	**1.083**	**1.228**	1.040	0.975	1.109
	Public 100 m	0.984	0.956	1.012	1.000	0.971	1.030	0.966	0.910	1.026	0.984	0.925	1.047
	Public 300 m	**0.968**	**0.941**	**0.996**	0.974	0.946	1.003	1.000	0.944	1.058	0.994	0.938	1.055
	Quiet 100 m	**0.943**	**0.918**	**0.969**	0.992	0.965	1.019	**.**	**.**	**.**	.	.	.
	Quiet 300 m	**0.922**	**0.898**	**0.946**	**0.960**	**0.934**	**0.986**	**.**	**.**	**.**	.	.	.
2019	Urban 100 m	**1.028**	**1.000**	**1.057**	**0.970**	**0.943**	**0.997**	**1.103**	**1.041**	**1.170**	1.027	0.967	1.090
	Urban300 m	1.017	0.990	1.046	**0.964**	**0.937**	**0.991**	**1.144**	**1.076**	**1.215**	1.053	0.989	1.120
	Public 100 m	**0.969**	**0.944**	**0.996**	0.986	0.959	1.014	0.948	0.896	1.004	0.971	0.915	1.029
	Public 300 m	**0.958**	**0.932**	**0.985**	**0.962**	**0.935**	**0.990**	1.000	0.946	1.057	0.996	0.941	1.055
	Quiet 100 m	**0.954**	**0.930**	**0.979**	0.994	0.968	1.021	**.**	**.**	**.**	**.**	**.**	**.**
	Quiet 300 m	**0.927**	**0.904**	**0.952**	**0.958**	**0.934**	**0.984**	**.**	**.**	**.**	**.**	**.**	**.**

* Statistically significant results (*p* < 0.005) are marked with bold text.

**Table A3:** Risk ratios for crude and multivariate associations between living in an apartment vs detached single-family house in availability to total urban green space (Urban), publicly available urban green space (Public) and quiet publicly available urban green space (Quiet). RR for 100 m and 300 m buffer distances from the residence for the three different types of urban green spaces are presented.

		Malmö City						Kristianstad City					
		Crude			Adjusted			Crude			Adjusted		
		RR	Lower	Upper	RR	Lower	Upper	RR	Lower	Upper	RR	Lower	Upper
2010	Urban 100 m	**1.798**	**1.749**	**1.848**	**1.909**	**1.848**	**1.972**	**3.329**	**3.096**	**3.579**	**2.124**	**1.918**	**2.354**
	Urban 300 m	**1.801**	**1.752**	**1.852**	**1.902**	**1.841**	**1.964**	**3.339**	**3.102**	**3.593**	**2.443**	**2.204**	**2.708**
	Public 100 m	**0.707**	**0.681**	**0.734**	**0.813**	**0.781**	**0.847**	**0.834**	**0.780**	**0.892**	**0.675**	**0.615**	**0.741**
	Public 300 m	**0.644**	**0.620**	**0.668**	**0.717**	**0.688**	**0.747**	**1.121**	**1.049**	**1.198**	0.951	0.866	1.044
	Quiet 100 m	**0.397**	**0.378**	**0.417**	**0.385**	**0.366**	**0.406**	**.**	**.**	**.**	**.**	**.**	**.**
	Quiet 300 m	**0.516**	**0.493**	**0.539**	**0.497**	**0.473**	**0.521**	**.**	**.**	**.**	**.**	**.**	**.**
2015	Urban 100 m	**1.692**	**1.642**	**1.744**	**1.828**	**1.766**	**1.892**	**2.308**	**2.173**	**2.452**	**1.820**	**1.672**	**1.980**
	Urban 300 m	**1.812**	**1.760**	**1.866**	**1.943**	**1.879**	**2.010**	**2.771**	**2.597**	**2.957**	**1.707**	**1.560**	**1.869**
	Public 100 m	**0.648**	**0.622**	**0.674**	**0.800**	**0.766**	**0.836**	**0.863**	**0.811**	**0.918**	**0.729**	**0.669**	**0.794**
	Public 300 m	**0.619**	**0.594**	**0.644**	**0.738**	**0.706**	**0.771**	1.040	0.982	1.102	**0.891**	**0.822**	**0.966**
	Quiet 100 m	**0.625**	**0.602**	**0.649**	**0.554**	**0.532**	**0.577**	**.**	**.**	**.**	**.**	**.**	**.**
	Quiet 300 m	**0.645**	**0.622**	**0.669**	**0.580**	**0.558**	**0.603**	**.**	**.**	**.**	**.**	**.**	**.**
2019	Urban 100 m	**1.828**	**1.774**	**1.882**	**1.998**	**1.934**	**2.064**	**2.309**	**2.177**	**2.450**	**2.250**	**2.085**	**2.428**
	Urban300 m	**1.852**	**1.799**	**1.906**	**2.051**	**1.986**	**2.118**	**2.632**	**2.473**	**2.802**	**2.107**	**1.945**	**2.283**
	Public 100 m	**0.643**	**0.619**	**0.669**	**0.785**	**0.753**	**0.818**	**0.813**	**0.766**	**0.863**	**0.726**	**0.673**	**0.782**
	Public 300 m	**0.633**	**0.609**	**0.659**	**0.742**	**0.711**	**0.774**	1.027	0.970	1.087	0.952	0.886	1.024
	Quiet 100 m	**0.609**	**0.587**	**0.633**	**0.562**	**0.540**	**0.584**	**.**	**.**	**.**	**.**	**.**	**.**
	Quiet 300 m	**0.659**	**0.635**	**0.683**	**0.621**	**0.597**	**0.645**	**.**	**.**	**.**	**.**	**.**	**.**

* Statistically significant results (*p* < 0.005) are marked with bold text.

**Table A4:** Risk ratios for crude and multivariate associations between living alone vs cohabiting in availability in availability to total urban green space (Urban), publicly available urban green space (Public) and quiet publicly available urban green space (Quiet). RR for 100 m and 300 m buffer distances from the residence for the three different types of urban green spaces are presented.

		Malmö City						Kristianstad City					
		Crude			Adjusted			Crude			Adjusted		
		RR	Lower	Upper	RR	Lower	Upper	RR	Lower	Upper	RR	Lower	Upper
2010	Urban 100 m	**0.792**	**0.772**	**0.812**	**0.893**	**0.868**	**0.918**	**0.626**	**0.583**	**0.673**	0.998	0.922	1.081
	Urban 300 m	**0.794**	**0.774**	**0.815**	**0.897**	**0.872**	**0.922**	**0.673**	**0.627**	**0.724**	**1.087**	**1.004**	**1.177**
	Public 100 m	**1.085**	**1.056**	**1.114**	0.996	0.968	1.025	**1.092**	**1.026**	**1.162**	1.052	0.982	1.127
	Public 300 m	**1.017**	**0.990**	**1.044**	**0.914**	**0.888**	**0.940**	1.012	0.949	1.079	**1.084**	**1.010**	**1.164**
	Quiet 100 m	**1.301**	**1.264**	**1.339**	**1.130**	**1.096**	**1.165**	**.**	**.**	**.**	**.**	**.**	**.**
	Quiet 300 m	**1.189**	**1.154**	**1.225**	**1.052**	**1.020**	**1.086**	**.**	**.**	**.**	**.**	**.**	**.**
2015	Urban 100 m	**0.784**	**0.762**	**0.806**	**0.854**	**0.829**	**0.880**	**0.777**	**0.732**	**0.825**	1.061	0.994	1.133
	Urban 300 m	**0.799**	**0.777**	**0.821**	**0.890**	**0.864**	**0.917**	**0.710**	**0.667**	**0.757**	1.044	0.974	1.119
	Public 100 m	**1.110**	**1.079**	**1.142**	0.998	0.968	1.029	1.060	0.999	1.124	1.013	0.950	1.080
	Public 300 m	**1.044**	**1.015**	**1.074**	**0.919**	**0.892**	**0.947**	0.979	0.926	1.036	0.995	0.936	1.058
	Quiet 100 m	**1.160**	**1.129**	**1.192**	**1.090**	**1.059**	**1.121**	**.**	**.**	**.**	.	.	.
	Quiet 300 m	**1.104**	**1.076**	**1.134**	1.018	0.990	1.047	**.**	**.**	**.**	.	.	.
2019	Urban 100 m	**0.793**	**0.772**	**0.816**	**0.873**	**0.848**	**0.899**	**0.786**	**0.742**	**0.834**	1.056	0.992	1.125
	Urban300 m	**0.813**	**0.791**	**0.835**	**0.895**	**0.869**	**0.921**	**0.734**	**0.690**	**0.780**	1.033	0.967	1.103
	Public 100 m	**1.118**	**1.088**	**1.149**	0.990	0.962	1.019	**1.074**	**1.016**	**1.137**	1.000	0.941	1.062
	Public 300 m	**1.048**	**1.019**	**1.077**	**0.921**	**0.894**	**0.948**	0.976	0.923	1.031	0.983	0.926	1.043
	Quiet 100 m	**1.157**	**1.127**	**1.187**	**1.090**	**1.061**	**1.121**	**.**	**.**	**.**	.	**.**	**.**
	Quiet 300 m	**1.117**	**1.089**	**1.146**	**1.038**	**1.010**	**1.067**	**.**	**.**	**.**	**.**	**.**	**.**

* Statistically significant results (*p* < 0.005) are marked with bold text.

**Table A5:** Risk ratios for crude and multivariate associations between born in Sweden vs born abroad in availability to total urban green space (Urban), publicly available urban green space (Public) and quiet publicly available urban green space (Quiet). RR for 100 m and 300 m buffer distances from the residence for the three different types of urban green spaces are presented.

		Malmö City						Kristianstad City					
		Crude			Adjusted			Crude			Adjusted		
		RR	Lower	Upper	RR	Lower	Upper	RR	Lower	Upper	RR	Lower	Upper
2010	Urban 100 m	**0.968**	**0.939**	**0.998**	**0.933**	**0.904**	**0.963**	0.936	0.833	1.051	**0.720**	**0.640**	**0.809**
	Urban 300 m	1.000	0.970	1.032	**0.964**	**0.934**	**0.995**	**0.867**	**0.773**	**0.971**	**0.678**	**0.604**	**0.760**
	Public 100 m	**0.877**	**0.851**	**0.905**	**0.946**	**0.916**	**0.976**	0.983	0.885	1.091	0.992	0.892	1.104
	Public 300 m	**0.872**	**0.846**	**0.899**	**0.948**	**0.919**	**0.978**	0.916	0.824	1.018	**0.895**	**0.805**	**0.996**
	Quiet 100 m	**0.950**	**0.919**	**0.982**	0.973	0.940	1.007	.	.	.	.	.	.
	Quiet 300 m	1.019	0.984	1.055	1.032	0.996	1.070	.	.	.	.	.	.
2015	Urban 100 m	**0.942**	**0.914**	**0.972**	**0.939**	**0.909**	**0.970**	0.993	0.903	1.092	**0.792**	**0.720**	**0.873**
	Urban 300 m	1.000	0.969	1.031	0.977	0.946	1.009	0.923	0.837	1.017	**0.679**	**0.615**	**0.750**
	Public 100 m	**0.781**	**0.757**	**0.805**	**0.883**	**0.856**	**0.912**	0.958	0.872	1.051	0.965	0.878	1.060
	Public 300 m	**0.800**	**0.776**	**0.825**	**0.902**	**0.874**	**0.931**	0.981	0.897	1.073	0.957	0.873	1.048
	Quiet 100 m	**1.142**	**1.107**	**1.178**	**1.087**	**1.053**	**1.123**	.	.	.	.	.	.
	Quiet 300 m	**1.173**	**1.137**	**1.209**	**1.133**	**1.097**	**1.169**	.	.	.	.	.	.
2019	Urban 100 m	**0.911**	**0.885**	**0.939**	**0.910**	**0.882**	**0.938**	0.931	0.858	1.009	**0.754**	**0.693**	**0.819**
	Urban300 m	**0.940**	**0.913**	**0.969**	**0.940**	**0.912**	**0.969**	0.887	0.816	0.964	**0.663**	**0.608**	**0.722**
	Public 100 m	**0.803**	**0.780**	**0.826**	**0.934**	**0.907**	**0.962**	0.983	0.908	1.065	0.982	0.905	1.066
	Public 300 m	**0.793**	**0.770**	**0.816**	**0.908**	**0.881**	**0.936**	0.941	0.871	1.017	**0.913**	**0.844**	**0.989**
	Quiet 100 m	**1.131**	**1.099**	**1.164**	**1.085**	**1.052**	**1.118**	.	.	.	.	.	.
	Quiet 300 m	**1.174**	**1.141**	**1.209**	**1.148**	**1.113**	**1.183**	.	.	.	.	.	.

* Statistically significant results (*p* < 0.005) are marked with bold text.

**Table A6:** Risk ratios for crude and multivariate associations between living in a high SES area vs low SES area in availability to total urban green space (Urban), publicly available urban green space (Public) and quiet publicly available urban green space (Quiet). RR for 100 m and 300 m buffer distances from the residence for the three different types of urban green spaces are presented.

		Malmö City						Kristianstad City					
		Crude			Adjusted			Crude			Adjusted		
		RR	Lower	Upper	RR	Lower	Upper	RR	Lower	Upper	RR	Lower	Upper
2010	Urban 100 m	**1.081**	**1.053**	**1.109**	**0.844**	**0.820**	**0.869**	**3.207**	**2.985**	**3.446**	**1.948**	**1.766**	**2.149**
	Urban 300 m	**1.093**	**1.065**	**1.121**	**0.851**	**0.826**	**0.876**	**2.947**	**2.742**	**3.167**	**1.674**	**1.518**	**1.846**
	Public 100 m	**0.715**	**0.696**	**0.735**	**0.767**	**0.745**	**0.790**	1.060	0.993	1.131	**1.387**	**1.271**	**1.513**
	Public 300 m	**0.704**	**0.685**	**0.723**	**0.772**	**0.749**	**0.795**	**1.247**	**1.167**	**1.332**	**1.318**	**1.205**	**1.442**
	Quiet 100 m	**0.919**	**0.894**	**0.946**	**1.168**	**1.134**	**1.203**	.	.	.	.	.	.
	Quiet 300 m	**0.947**	**0.920**	**0.974**	**1.130**	**1.096**	**1.166**	.	.	.	.	.	.
2015	Urban 100 m	0.977	0.950	1.004	**0.783**	**0.758**	**0.808**	**2.168**	**2.042**	**2.301**	**1.494**	**1.377**	**1.620**
	Urban 300 m	**1.039**	**1.011**	**1.068**	**0.811**	**0.786**	**0.837**	**2.983**	**2.795**	**3.183**	**2.165**	**1.983**	**2.363**
	Public 100 m	**0.601**	**0.583**	**0.619**	**0.656**	**0.635**	**0.677**	1.048	0.986	1.114	**1.304**	**1.202**	**1.415**
	Public 300 m	**0.614**	**0.597**	**0.632**	**0.673**	**0.652**	**0.695**	**1.165**	**1.100**	**1.234**	**1.263**	**1.168**	**1.365**
	Quiet 100 m	**1.210**	**1.178**	**1.242**	**1.390**	**1.351**	**1.430**	.	.	.	.	.	.
	Quiet 300 m	**1.146**	**1.117**	**1.176**	**1.282**	**1.247**	**1.318**	.	.	.	.	.	.
2019	Urban 100 m	**0.904**	**0.879**	**0.929**	**0.743**	**0.721**	**0.766**	**1.728**	**1.630**	**1.833**	**1.132**	**1.052**	**1.217**
	Urban300 m	**0.895**	**0.871**	**0.920**	**0.727**	**0.705**	**0.749**	**2.319**	**2.179**	**2.469**	**1.601**	**1.481**	**1.730**
	Public 100 m	**0.566**	**0.550**	**0.582**	**0.606**	**0.588**	**0.625**	**1.029**	**0.972**	**1.089**	**1.233**	**1.151**	**1.322**
	Public 300 m	**0.601**	**0.584**	**0.618**	**0.648**	**0.629**	**0.668**	**1.108**	**1.048**	**1.172**	**1.149**	**1.073**	**1.231**
	Quiet 100 m	**1.190**	**1.160**	**1.221**	**1.322**	**1.286**	**1.359**	.	.	.	.	.	.
	Quiet 300 m	**1.110**	**1.082**	**1.139**	**1.189**	**1.157**	**1.222**	.	.	.	.	.	.

* Statistically significant results (*p* < 0.005) are marked with bold text.
